# Efficiency measurement and spatial spillover effect of provincial health systems in China: Based on the two-stage network DEA model

**DOI:** 10.3389/fpubh.2022.952975

**Published:** 2022-10-03

**Authors:** Yuping Yang, Liqin Zhang, Xiaoyan Zhang, Mengting Yang, Wenjie Zou

**Affiliations:** School of Economics, Fujian Normal University, Fuzhou, China

**Keywords:** health system, two-stage network DEA model, resource allocation efficiency, service operation efficiency, spatial spillover effect

## Abstract

The effectiveness of a health care system is an important factor for improving people's health and quality of life. The purpose of this research is to analyze the efficiency and spatial spillover effects of provincial health systems in China using panel data from 2009 to 2020. We employ the two-stage network DEA model to evaluate their efficiencies and use a spatial econometric model for empirical estimation. The results suggest that the overall efficiency, resource allocation efficiency, and service operation efficiency of health systems in different regions of China generally have fluctuating upward trends, with large differences in efficiency among the various regions. Further analysis reveals that the efficiency of China's health system has a significant spatial spillover effect. The level of economic development, fiscal decentralization and old-age dependency ratio are important factors affecting the health system efficiency. Our findings help to identify the efficiency and internal operating mechanisms of China's health system at different stages, and are expected to contribute to policymakers' efforts to build a high-quality health service system.

## Introduction

As it relates to people's health, public health is an important area for improving people's quality of life and promoting economic development. Insufficient or inefficient spending on public health reduces the health of citizens, which in turn slows down the process of economic development (1). The outbreak of the COVID-19 pandemic has had a great impact on the public health system of countries, seriously threatening the lives of many people (2). Therefore, in the context of COVID-19 ravaging the world, how to effectively improve health system efficiency and protect people's health has become a major issue that needs to be solved urgently.

Since the reform of its medical and health system in 2009, China has been committed to improving its national public health system and continuously helping to better the health and physical quality of the people. The 18th National Congress of the Communist Party of China released the Healthy China 2030 Plan, which explicitly calls for building a sound medical security system and enhancing the quality of health services, thereby meeting the public's demand for health. In terms of public health expenditure, China's fiscal expenditure on medical and health rose from 399.419 billion yuan in 2009 to 1921.619 billion yuan in 2020, accounting for 7.82% of the government's public budget expenditure from 5.23%. Clearly, the central government attaches great importance to basic medical and health services and continues to improve the quantity and quality of public health supplies. However, the situation of health care in China is not optimistic. As the disease spectrum changes and population aging intensifies, China's total health resources remain inadequate. The problems of difficult medical treatment and high cost for patients have not been solved, and the imbalance of health resource allocation among regions is severe. Moreover, the outbreak of the COVID-19 pandemic has posed a serious challenge to the supply capacity of its health system. It is foreseeable that the China government will focus on increasing the supply of health resources and improving health system efficiency in the long run.

Health system efficiency is an important indicator of how well a health system is functioning (3). Although a comprehensive estimation of such efficiency is a complex task (4), it can effectively measure the allocation of health resources and the level of residents' health output. Currently, most studies use parametric and non-parametric methods to evaluate health system efficiency. Among them, non-parametric methods are represented by Data Envelopment Analysis (DEA) (3, 5), and parametric methods are mainly represented by Stochastic Frontier Analysis (SFA) (6, 7). Some scholars have combined the two to study health care efficiency (8), but SFA may lead to biased measurements due to its dependence on the functional form and the distribution of random errors (9). In addition, most studies apply DEA to assess health system efficiency by considering the health system as a “black box,” but fail to focus on the internal operating patterns and variability characteristics of the health system. Although a few studies in the literature have used a two-stage DEA model to estimate health expenditure efficiency, the whole production process and the two sub-stages are regarded as independent of each other, which does not reflect the connection between different stages and the overall process. Therefore, this paper adopts the two-stage network DEA model to measure health system efficiency in China from 2009 to 2020 and reveals the operation status of health systems in different regions at different stages. We look to answer the following questions. What are the differences in health system efficiency among the regions in China and the reasons for them? Are there spatial spillover effects in health system efficiency across regions? What should be done to improve the efficiency of China's health system?

The main contributions of this study are summarized as follows. First, we divide a health system into resource allocation stage and service operation stage and adopt the two-stage network DEA model to measure health system efficiency, so as to reveal the differential characteristics of the system's different stages. Second, this paper provides an in-depth analysis of the evolutionary trends of efficiency at different stages of the health system, while visualizing health system efficiency by combining relevant contents of geography, and then comprehensively examines the spatial evolutionary characteristics of health system efficiency in China. Third, most studies in the literature only focus on the efficiency changes and influencing factors of a local health system, but lack any analysis of the impacts of inter-regional health systems, and so they do not reveal these systems' spillover effects and influence mechanisms. Therefore, this paper further analyzes the spatial spillover effect of health system efficiency and extensively explores the influencing factors of health system efficiency and their spatial spillover effects in terms of economic development, fiscal decentralization, population structure, and education level, so as to provide a useful addition to the existing literature.

The remaining contents of this paper are arranged as follows. The Literature Review section mainly reviews the relevant literature. The Methods section introduces the research methods, variable measurements and data. The Empirical Analysis section discusses the empirical results. The Conclusion and Policy Recommendations section summarizes the research conclusions and puts forward relevant policy recommendations.

## Literature review

### Health system efficiency

Scholars have conducted a large number of studies in the field of health. Especially after the COVID-19 pandemic, health system efficiency has become a greater focus of scholars and policy makers.

The existing research on health system efficiency mainly focuses on the application and measurement of health system efficiency. The non-parametric method based on data envelopment analysis has become the mainstream and is the most commonly used efficiency evaluation technology in the health field. In 1983, Nunamaker (10) first applied DEA method to the research in the field of medical and health care. Subsequently, Banker et al. (11) also used this method to evaluate the multivariate input-output efficiency of American teaching hospitals. Since then, the number of studies using DEA model to measure different types of medical and health institutions has been increasing, and the research objects include primary medical and health institutions, hospitals, professional public health institutions, and public health systems. For example, Tsai and Molinero (12) estimated and analyzed the efficiency of 27 NHS institutions in the UK based on the non-parametric DEA method. Kontodimopoulos et al. (13) evaluated the operation efficiency of 17 small-scale hospitals in rural Greece. Novignon (14) applied DEA model to investigate the efficiency of health expenditure in sub-Saharan Africa and found that the efficiency of local health expenditure was low.

With the deepening of research, the deficiencies of the traditional DEA model have become increasingly prominent. Many studies have improved and optimized the traditional DEA model and gradually applied it to various fields of economy and society. On this basis, the measurement of health system efficiency has been further expanded, gradually evolving from the application of the traditional DEA model to the use of a more efficient and comprehensive DEA model. It mainly includes Super-SBM model (15, 16), Bootstrap DEA model (17, 18), Dynamic Network DEA model (19). Some studies also adopt the Malmquist productivity index to decompose healthcare efficiency (15, 20). Meanwhile, a three-stage DEA model is employed in the empirical analysis (21, 22). Moreover, more and more scholars are no longer limited to using the DEA model alone, they tend to combine the DEA model with other models to evaluate the health system efficiency. For example, Rouyendegh et al. (23) combined the fuzzy analytic hierarchy process with DEA to quantify the data and construct a DEA-FAHP model.

In recent years, people's health has been greatly threatened by the increasingly serious pollution problem, which poses a challenge to the operation of the health system. Therefore, scholars have begun to attach importance to the evaluation of health expenditure efficiency in combination with environment and public health (24), so as to more comprehensively investigate the real situation of health system efficiency. Some studies put energy consumption, environmental pollution and public health in the same framework, and analyzed the efficiency values at different stages by constructing a dynamic network DEA model (25–27). These studies divide the whole model into production stage and health treatment stage. The output of the production stage mainly includes pollutants, which are regarded as the input of the health treatment stage, and the output of the health treatment stage involves indicators related to residents' health such as disease incidence. For example, Chen et al. (25) integrated pollution, energy, public health, and social media, adopted a modified Undesirable Dynamic Network model to analyze the efficiency of different stages, and discovered that the production stage of cities in China is more efficient than the health treatment stage, and that the disparity in sanitation expenditures among the cities is large and inefficient.

After the outbreak of COVID-19 pandemic, scholars have increased relevant research in the field of health care, mainly concerning the operational efficiency of health institutions (28), health expenditure efficiency (2, 29) and government public health management efficiency (30, 31). For example, Kamel and Mousa (28) used the DEA model to measure the operational efficiency of 26 isolation hospitals in Egypt during the COVID-19 pandemic and identified important drivers affecting their efficiency. Martínez-Córdoba et al. (31) calculated the efficiency of government public health resource management in the context of the COVID-19 pandemic.

In addition, the influencing factors of health efficiency have also been widely concerned. Previous studies have mainly analyzed the influencing factors of health system efficiency from many aspects, including economic conditions, government level, social and environmental factors. From the perspective of economic status, most scholars' studies have shown that economic status is an important factor affecting the health system efficiency (32, 33). At the government level, official corruption and policy formulation have a significant impact on the health spending efficiency (14, 34–36). In terms of social and environmental factors, health system efficiency is also constrained by demographics, ethnic characteristics, and environmental performance (34, 37). In recent years, an increasing number of studies have focused on the impact of institutional factors on health systems efficiency, especially the effect of fiscal decentralization (38, 39). For example, Zhou (39) proposed that fiscal decentralization reduces the efficiency of health spending in China by distorting government spending decisions and is a major factor affecting the functioning of the health system.

### Spatial spillover effects

In recent years, there has been an increasing focus on the interactions between economic agents and the correlation between things, which requires explicit consideration of spatial factors. LeSage and Pace (40) regarded the spatial spillover effect as one of the core contents of spatial econometric model estimation, and believed that the spatial spillover effect was a measure of the impact of a variable change in a single region on other regions. On this basis, the research on spatial spillover effect has developed rapidly, and the focus of the research has evolved from focusing on macro-economic growth issues to micro-environmental welfare.

On the one hand, the spatial spillover effect is applied to the study of economic growth. Previous literatures mostly focus on the spillover effects of economic growth from the perspective of specific industries, involving the fields of transportation infrastructure and tourism (41–43). For example, Hu and Liu (41) used a spatial econometric model to examine the existence of positive externalities of transportation and its spillover effects on economic growth in adjacent regions. Tong et al. (42) argued that the spillover effect of road infrastructure on agricultural output in neighboring states varies with the spatial weight matrix used in the model. Ma et al. (43) pointed out that the economic growth effect produced by tourism development has the characteristics of spatial spillover. In recent years, some studies have also conducted in-depth analysis of the spillover effects of economic growth from different perspectives (44, 45).

On the other hand, the spatial spillover effect is employed to the research of environmental pollution. With the increasingly serious global environmental pollution problem, scholars have begun to explore the characteristics of pollution problems from different angles. In this context, the spatial spillover effect has been widely used in the study of pollution emissions (46), and scholars are committed to finding the key to solving the environmental pollution problem from the perspective of spatial correlation, including urbanization (47), environmental regulation (48, 49), and financial development (50, 51). For example, Li et al. (47) explored the spatial spillover effects of industrialization and urbanization on the discharge of seven pollutants in the Huang-huai-hai region of China based on the spatial Durbin model. Feng et al. (48) emphasized the spatial spillover effect of environmental regulation on PM2.5 concentration, and identified the influencing factors of pollution spillover, such as industrial structure and population density. Zhong and Li (50) explored the relationship between financial development and green total factor productivity and its spatial spillover effects. Khezri et al. (51) discussed the direct and spillover effects of financial development on CO2 emissions, confirming the importance of the influence of neighboring countries on their own CO_2_ emissions. In addition, there are also some literatures that incorporate both economic growth and environmental issues into the research framework, and analyze the spatial spillover effects of green economic growth.

## Methods

### Two-stage network DEA model

Data Envelopment Analysis (DEA) can deal with the problem of multiple inputs and multiple outputs. As such, it has been continuously expanded and improved in the development process and has been widely used to evaluate the relative efficiency of decision-making units. Traditional DEA is an important method for evaluating efficiency. The existing literature applies the model to analyze problems in different areas of the economy and society, and provides many useful insights. These studies mainly involve the evaluation of the efficiency of financial institutions. Sufian and Kamarudin (52) measured the profit efficiency of individual banks operating in Bangladesh by using the Slack-Based DEA, and the findings provided empirical evidence on the level of profit efficiency in the country's banking sector. Aghimien et al. (53) employed the DEA to assess the technical efficiency, scale efficiency and pure technical efficiency of Gulf Cooperation Council banks. Kamarudin et al. (54) used the Slack Based DEA model to calculate the profit efficiency of 31 commercial banks operating in Bangladesh from 2004 to 2011 and investigated the determinants of bank profit efficiency. Hussain et al. (55) evaluated the level of bank revenue efficiency based on the Non-parametric DEA method. In addition, a large number of studies have also used traditional DEA model to assess energy efficiency and environmental performance (56–59).

However, traditional DEA models treat the decision-making unit as a “black box” and fail to pay attention to the internal structure of the system, which may lead to misleading results (60, 61). In contrast, the two-stage network DEA model is an extension of the traditional DEA model, and its advantages are mainly reflected as follows. On the one hand, it analyzes the organizational structure contained in the decision-making unit (60). By dividing the decision-making unit into different production processes, and using input elements, output elements and intermediate variables to closely link the different production processes of the decision-making unit, so as to reflect the logical relationship of input and output in different stages. On the other hand, the two-stage network DEA model helps to improve the accuracy of measurement results and specifically analyze the sources of system inefficiencies, thereby obtaining richer information (62). Through the analysis of the sub-stage and the overall stage, the operation of the complex production system can be effectively identified and the efficiency of each sub-process can be evaluated, and then reveal the ineffective source of the system and the different characteristics of different stages.

Two-stage DEA assumes that the entire production system is composed of two sub-stages. Stage 1 uses initial input *X* to produce intermediate output *Z*, and stage 2 uses intermediate output *Z* to produce final output *Y*. In order to calculate the expected production frontier in the entire system, the output of stage 1 is required to be exactly the expected input of stage 2.

Assuming that there are *n* decision making units (DMU), each DMU has *m* input terms and *s* output terms. *X*_*ij*_ is the *i*^th^ input of the *j*^th^ DMU, expressed as *X*_*j*_ = (*x*_1*j*_, *x*_2*j*_,…, *x*_*mj*_)^T^; *Y*_*rj*_ is the *r*^th^ output of the *j*^th^ DMU, expressed as *Y*_*j*_ = (*y*_1*j*_, *y*_2*j*_,…, *y*_*sj*_)^T^; *Z*_*pj*_ is the *p*^th^ intermediate output of the *j*^th^ DMU, expressed as *Z*_*j*_ = (*z*_1*j*_, *z*_2*j*_,…, *z*_*qj*_)^T^. Under the premise of constant returns to scale, the formula for measuring the efficiency of the *k*^th^ DMU is as follows:


(1) $$\begin{array}{l} E_{k}=\max\;\sum_{r=1}^{s}u_{r}Y_{rk}/\sum_{i=1}^{m}v_{i}X_{ik}\\ s.t.\;\sum_{r=1}^{s}u_{r}Y_{rj}/\sum_{i=1}^{m}v_{i}X_{ij}\;\leq 1,\;j=1,2,\ldots ,n\\ u_{r},v_{i}\geq \varepsilon ,\;r=1,2,\ldots ,s;\;i=1,2,\ldots ,m \end{array}$$

among which, the efficiency of stage 1 is:


(2) $$\begin{array}{l} E_{k}^{1}=\max\;\sum_{p=1}^{q}w_{p}Z_{pk}/\sum_{i=1}^{m}v_{i}X_{ik}\\ s.t.\;\sum_{p=1}^{q}w_{p}Z_{pj}/\sum_{i=1}^{m}v_{i}X_{ij}\leq 1,\;j=1,2,\ldots n\\ w_{p},v_{i}\geq \varepsilon ,\;p=1,2,\ldots q;\;i=1,2,\ldots m \end{array}$$

And the efficiency of stage 2 is:


(3) $$\begin{array}{l} E_{k}^{2}=\;\max\sum_{r=1}^{s}u_{r}Y_{rk}/\sum_{p=1}^{q}w_{p}Z_{pk}\\ s.t.\;\sum_{r=1}^{s}u_{r}Y_{rj}/\sum_{p=1}^{q}w_{p}Z_{pj}\leq 1,j=1,2,\ldots ,n\\ u_{r},w_{p}\geq \varepsilon ,\;r=1,2,\ldots ,s;\;p=1,2,\ldots ,q \end{array}$$

Here, ε is a small non-Archimedean number; ***v*** = (*v*_1_*, v*_2_*, …, v*_*m*_)^T^ is the coefficient vector of input *X*; ***u*** = (*u*_1_*, u*_2_*, …, u*_*s*_)^T^ is the coefficient vector of final output *Y*; and ***w*** = (*w*_1_*, w*_2_*, …, w*_*q*_)^T^ is the coefficient vector of intermediate output *Z*. *E*_*k*_ = 1 indicates that DEA is valid, and *E*_*k*_ < 1 indicates that DEA is invalid.

By solving Equations (1), (2), and (3), the overall efficiency *E*_*k*_, stage 1 efficiency $E_{k}^{1}$, and stage 2 efficiency $E_{k}^{2}$ can be obtained, respectively. However, the efficiency of this traditional two-stage DEA measure is performed independently and cannot link the different stages and the overall process. Drawing on the research of Kao and Hwang (60), this study integrates the traditional two-stage DEA model and converts it into a linear equivalent model as follows:


(4) $$\begin{array}{l} E_{k}=\max\;\sum_{r=1}^{s}u_{r}Y_{rk}\\ s.t.\;\sum_{i=1}^{m}v_{i}X_{ik}=1,\\ \sum_{r=1}^{s}u_{r}Y_{rj}-\sum_{i=1}^{m}v_{i}X_{ij}\leq 0,\;j=1,2,\ldots ,n\\ \sum_{p=1}^{q}w_{p}Z_{pj}-\sum_{i=1}^{m}v_{i}X_{ij}\leq 0,\;j=1,2,\ldots ,n\\ \sum_{r=1}^{s}u_{r}Y_{rj}-\sum_{p=1}^{q}w_{p}Z_{pj}\leq 0,\;j=1,2,\ldots ,n\\ u_{r},\;v_{i},\;w_{p}\geq \varepsilon ,\;r=1,2,\ldots ,s;\;i=1,2,\ldots ,m;\;p=1,2,\ldots ,q \end{array}$$

At this time, $E_{k}=E_{k}^{1}\times E_{k}^{2}$. However, the optimal solution obtained from Equation (4) may not be unique, and decomposition of the overall efficiency value is not guaranteed to be unique. Therefore, Kao and Hwang (60) proposed to find a set of multiplied subsets that generate the maximum efficiency value while still solving for the overall efficiency value according to Equation (4). Details are as follows:


(5) $$\begin{array}{l} E_{k}^{1}=\max\sum_{p=1}^{q}w_{p}Z_{pk}\\ s.t.\;\sum_{i=1}^{m}v_{i}X_{ik}=1\\ \sum_{r=1}^{s}u_{r}Y_{rk}-E_{k}\sum_{i=1}^{m}v_{i}X_{ik}=0\\ \sum_{r=1}^{s}u_{r}Y_{rj}-\sum_{i=1}^{m}v_{i}X_{ij}\leq 0,\;j=1,2,\ldots ,n\\ \sum_{p=1}^{q}w_{p}Z_{pj}-\sum_{i=1}^{m}v_{i}X_{ij}\leq 0,\;j=1,2,\ldots ,n\\ \sum_{r=1}^{s}u_{r}Y_{rj}-\sum_{p=1}^{q}w_{p}Z_{pj}\leq 0,\;j=1,2,\ldots ,n\\ u_{r},v_{i},w_{p}\geq \varepsilon ,r=1,2,\ldots ,s;\;i=1,2,\ldots ,m;\;p=1,2,\ldots ,q \end{array}$$

The efficiency value of stage 1 can be solved by Equation (5), and the efficiency value of stage 2 can be obtained by $E_{k}^{2}=E_{k}/E_{k}^{1}$. Similarly, the maximum efficiency of stage 2 can be solved first, and then the efficiency of stage 1 can be calculated.

### Spatial autocorrelation

Spatial autocorrelation is an index used to measure the degree of spatial agglomeration of an attribute in a region. It is mainly divided into global spatial autocorrelation and local spatial autocorrelation. Global spatial autocorrelation mainly examines the spatial correlation degree of an attribute in the whole area, which is usually tested by Moran's *I* index. The calculation formula is as follows:


(6) $$\begin{array}{l} \text{Mora}{\text{n}}^{\prime }\text{s }I=\frac{\sum_{i=1}^{n}\sum_{j=1}^{n}W_{ij}(x_{i}-\bar{x})(x_{j}-\bar{x})}{S^{2}\sum_{i=1}^{n}\sum_{j=1}^{n}W_{ij}}\\ \;S^{2}=\frac{\sum_{i=1}^{n}(x_{i}-\bar{x}{)}^{2}}{n} \end{array}$$

where *n* denotes the total number of units in the study; *W*_*ij*_ denotes the spatial weight matrix; *x*_*i*_ and *x*_*j*_ respectively, denote the observed values in spatial units *i* and *j*; and $\bar{x}$ denotes the mean of all spatial units. The value range of Moran's *I* index is [−1, 1]. When the value is >0, it indicates positive spatial correlation; when it is <0, it indicates negative spatial correlation; when it is equal to 0, it indicates random distribution in space (i.e., no correlation).

### Spatial econometric model

Compared with traditional linear regression models, spatial econometric models can effectively address the issues of complex spatial dependence and spatial correlation (63). At present, common spatial econometric models mainly include spatial lag model (SLM), spatial error model (SEM), and spatial Durbin model (SDM). Among them, SDM considers the spatial dependence of independent and dependent variables at the same time, so that the estimation results are not affected by the degree of spatial dependence of omitted variables. It is an optimization of the first two models and has a wider application (64). Therefore, this study adopts the spatial Durbin model to analyze the influencing factors of health system efficiency in China. The specific form of the model is as follows.


(7) $$\begin{array}{l} Y=\alpha +\rho WY+\beta X+\eta WX+\mu +v+\varepsilon \end{array}$$

where *Y* denotes health system efficiency; ρ denotes the spatial autoregressive coefficient; *W* denotes the spatial weight matrix; *X* denotes the factors that affect health system efficiency; β denotes the regression coefficients of influencing factors; η denotes the coefficients of spatial lag items in influencing factors; μ and *v* denote individual fixed effects and time fixed effects, respectively; and ε denotes the stochastic error.

### Variable selection and data source

#### Input-output indicators

**Figure 1:**
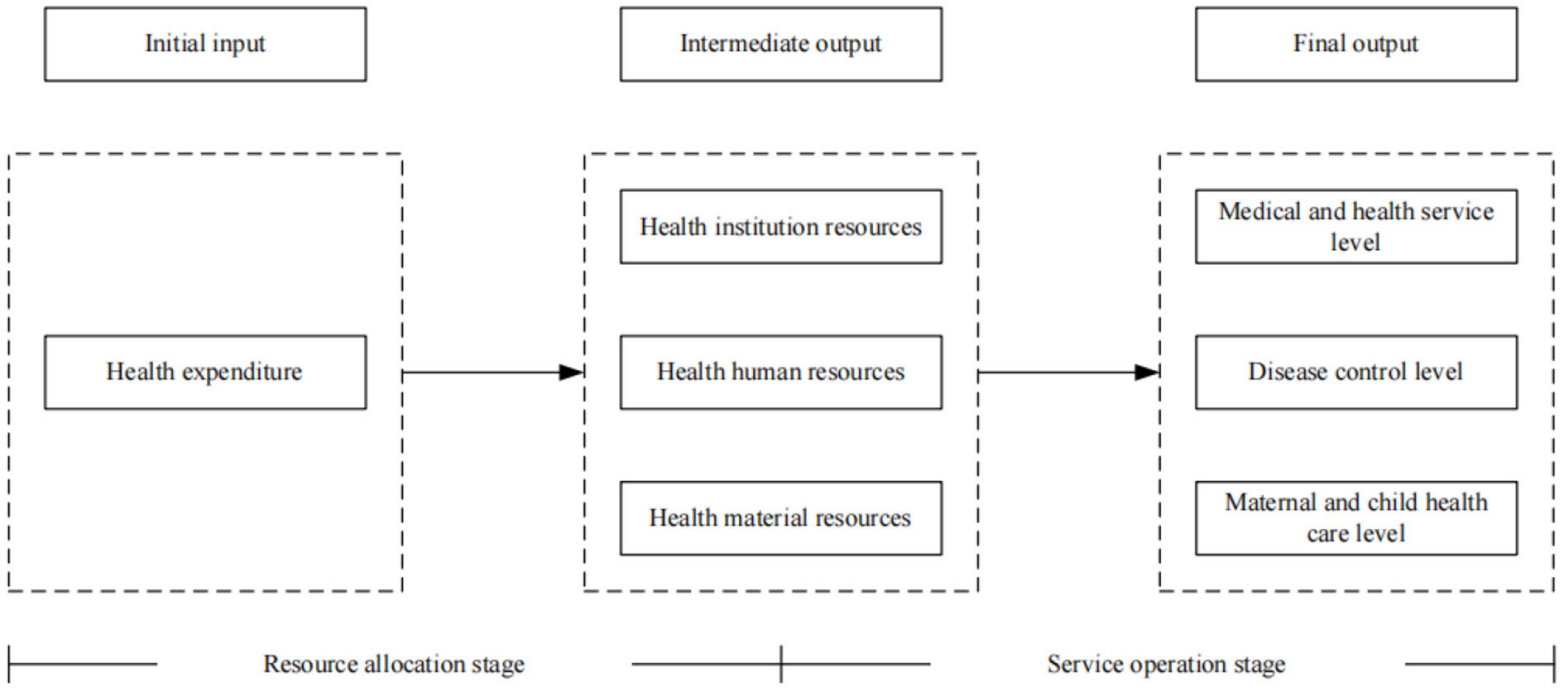
The production process of a health system.

According to the characteristics of the operations of the medical and health system, the health production process of the health system is regarded as a multi-stage value transfer process (65). Health financing is the source of the health system. It forms available health resources by investing in health funds, which in turn determines the utilization of health services and the health level of residents (66). Guo et al. (65) believed that the whole process of the health system from initial resource input to final goal realization actually includes two stages: resource allocation and service operation. Among them, the resource allocation stage refers to the process of producing a certain quantity and quality of health resources by investing health funds. The service operation stage refers to the process in which existing health resources serve patients and produce certain social benefits. Therefore, referring to the research of Guo et al. (65), we divide the health production process of the health system into two stages: resource allocation and service operation, as shown in Figure 1. We name the efficiency value measured in the resource allocation stage as resource allocation efficiency, that is - the first stage efficiency. At the same time, the efficiency value measured in the service operation stage is named service operation efficiency, that is - the second-stage efficiency.

At present, most studies regard the input-output process of the health system as a “black box” when evaluating health efficiency, and fail to consider the intermediate production process and the different characteristics of each stage. Therefore, in the selection of input indicators, human, financial and material resources are all regarded as input indicators of health efficiency. However, for the medical and health system, there is a sequential relationship between financial resources, material resources and human resources. Health expenditures are largely used for the construction of health infrastructure, including the establishment of health institutions, the purchase of equipment, and the introduction of health technicians (67). Therefore, in the resource allocation stage, we refer to the research of Zhou (39) to choose health expenditure as the only input indicator, and use the per capita medical and health expenditure to measure it. Health institution resources, health human resources, and health material resources are selected as output indicators, and the output indicators are measured by the number of medical and health institutions per thousand population, health technicians per thousand population, and beds in health institutions per thousand population, respectively.

In the service operation stage, the purpose of health resources investment is to improve the level of medical services and residents' health (66). Therefore, we regard the output of health resources in the previous stage as the input index of this stage, and select the level of medical and health services and residents' health as the final health output index. From the perspective of medical and health services, the service items of medical institutions focus on diagnosis and treatment services and inpatient services (68). Therefore, the level of medical and health services is measured by the number of diagnoses and treatments and hospital bed utilization rate (39, 68). From the perspective of residents' health output, Yu et al. (9) pointed out that the health output of residents can be measured from the aspects of disease control and maternal and child health care. Therefore, we select the incidences of class A and B notifiable infectious diseases to measure the level of disease control, and select the maternal mortality and perinatal mortality to measure the level of maternal and child health care. This is because the incidence of infectious diseases, maternal mortality and perinatal mortality describe the health output status of residents from different perspectives, and panel data are easy to obtain (9). Since the incidence of infectious diseases, maternal mortality, and perinatal mortality are negative indicators, we carried out positive processing of these three indicators, that is - taking the reciprocal. The specific input-output indicators system involved in health system efficiency appears in Table 1.

**Table 1:** Input-output indicators of health system efficiency.

**Category**	**Dimension**	**Specific indicators**
Initial investment	Health expenditure	Per capita medical and health expenditure (*I1*)
Intermediate output	Health institution resources	Number of health care institutions per thousand population (*M1*)
	Health human resources	Health technicians per thousand population (*M2*)
	Health material resources	Number of beds in health institutions per thousand population (*M3*)
Final output	Medical and health service level	Number of diagnoses and treatments (*F1*)
		Hospital bed utilization rate (*F2*)
	Disease control level	Incidence of class A and B notifiable infectious diseases (*F3*)
	Maternal and child health care level	Maternal mortality (*F4*)
		Perinatal mortality (*F5*)

#### Influencing factors

Existing studies have pointed out that the health system efficiency is affected by many factors such as economic conditions, institutional conditions, demographic characteristics and social environment (39, 68). On this basis, combined with the review and summary of the existing literature, we analyze the influencing factors of the health system efficiency from the aspects of economic status, institutional factors, population structure and education level. From the perspective of economic status, a country's economic environment directly determines the input intensity of its health resources, and then affects the supply capacity and quality of medical and health services (69). Commonly used measurement indicators include economic development level and urbanization rate. The level of economic development is the main factor restricting the supply of basic medical and health resources in a region, which reflects the economic strength and the level of people's welfare in a region (15). Therefore, it must be added to the model. We use per capita GDP of each region to assess economic development level (*pgdp*). Urbanization plays an important role in medical expenditure, provision of public goods and facilities, and utilization of health services (39, 70, 71). Liang (72) believed that the urbanization rate reflects the public health level of a region. Therefore, this study uses the proportion of urban population to the total population of each region at the end of the year to measure urbanization rate (*urban*).

From the perspective of institutional factors, China's fiscal decentralization system enables local governments to have greater power to allocate resources, which further reflects the ability of local governments in providing public services. Most scholars emphasized that the fiscal decentralization system affects the decision-making of local governments in the provision of health services, which in turn affects the health care efficiency (73, 74). Therefore, we incorporate fiscal decentralization (*fiscal*) into the analysis model, and use the proportion of local government per capita fiscal expenditure to central government per capita fiscal expenditure to measure it. From the perspective of population structure, changes in population structure are also a key factor affecting the effect of health care expenditures (71, 75). At present, the aging phenomenon of China's population is gradually becoming prominent, which undoubtedly has a certain effect on the operation efficiency of the health system. In this study, we use the old-age dependency ratio (*depend*) to characterize the changes in the population structure, and select the dependency ratio of the elderly population in each region to express it. From the perspective of education level, many studies have confirmed the close relationship between education level and health care efficiency (9, 73), which means that education level needs to be included in the regression model. We use the illiteracy rate (*edu*) as a proxy for education level, and choose the proportion of illiterate population to the population aged 15 and above in each region for evaluation. A lower proportion of illiterate population means a higher level of education.

#### Data sources

In view of data availability, we choose panel data of 31 provinces in China from 2009 to 2020 (excluding Hong Kong, Macao and Taiwan). The data of all indicators are obtained from China Statistical Yearbook, China Health Statistical Yearbook, China Population and Employment Statistics Yearbook, and statistical yearbooks of various provinces.

To sum up, Tables 2, 3 report the descriptive statistical analysis results of input-output indicators and regression variables, respectively.

**Table 2:** Descriptive statistics of input-output indicators.

**Variable**	**Obs**	**Mean**	**Std. Dev**.	**Min**	**Max**
*I1*	372	911.9729	520.6888	199.8247	3944.5350
*M1*	372	0.7528	0.3270	0.2018	2.1767
*M2*	372	5.8895	1.5395	2.7355	12.6152
*M3*	372	4.9666	1.1944	2.6047	7.9894
*F1*	372	23650.07	18615.05	959.00	89200.00
*F2*	372	83.7796	7.7772	48.3000	100.2000
*F3*	372	0.0046	0.0017	0.0015	0.0124
*F4*	372	0.0975	0.1003	0.0043	0.9091
*F5*	372	1.9618	0.8470	0.4160	5.5556

**Table 3:** Descriptive statistics of regression variables.

**Variable**	**Obs**	**Mean**	**Std. Dev**.	**Min**	**Max**
*efficiency*	372	0.5775	0.1735	0.1478	1.0000
*pgdp*	372	45846.53	24188.58	10971.00	128207.00
*fiscal*	372	7.0400	3.8179	2.6793	24.3355
*urban*	372	0.5673	0.1370	0.2230	0.8960
*edu*	372	6.1046	6.1594	0.8900	41.1800
*depend*	372	13.9660	3.6932	6.7100	25.4800

## Empirical analysis

### Evolution of temporal and spatial pattern of health system efficiency in China

#### Measurement results of health system efficiency in China

According to the above research methods, we obtain the efficiency values of the two-stage health system for each province in different years. In order to compare and analyze health system efficiency between different provinces, this study calculates the average health system efficiency of 31 provinces in China from 2009 to 2020 and reports the results in Table 4. In terms of overall efficiency, the level of health systems in most provinces in China is relatively low. For example, the average overall efficiency of health systems in Beijing, Inner Mongolia, Hainan, Guizhou, Tibet, Qinghai and other provinces is <0.5. Most of these provinces are located in the western region, and the backward level of economic development is the main reason for the low overall efficiency of the health system in the western region. On the contrary, although Beijing and Shanghai are located in the developed eastern region, the overall efficiency of their health systems is lower, only 0.3210 and 0.5255, respectively. This is because Beijing and Shanghai have a high level of economic development and rich medical resources, which have a strong siphon effect on the labor force and medical demanders, resulting in an overload of the medical system, thus reducing the efficiency of the regional health system. Most studies have confirmed this view (39, 65, 76). In comparison, the overall efficiency of the health system in the eastern and central regions is relatively high, with Shandong, Henan, Jiangsu, and Hebei having the highest overall efficiency. The reason may be that the geographical distance between Shandong and Hebei and regions with rich medical resources is relatively short, medical patients tend to transfer to adjacent developed areas to obtain better medical resources, and the net output of medical patients helps reduce the burden of regional health system, so as to alleviate the dilemma of shortage of health resources (39).

**Table 4:** Average efficiency of health system at different stages in each province.

**Province**	**Overall efficiency**	**Resource allocation**	**Service operation**	**Province**	**Overall efficiency**	**Resource allocation**	**Service operation**
Beijing	0.3210	0.5617	0.5686	Hubei	0.6724	0.7711	0.8710
Tianjin	0.5076	0.5672	0.8930	Hunan	0.7170	0.8483	0.8453
Hebei	0.7510	0.7815	0.9585	Guangdong	0.7168	0.7168	1.0000
Shanxi	0.5693	0.8644	0.6610	Guangxi	0.6162	0.6805	0.9059
Inner Mongolia	0.4041	0.6399	0.6327	Hainan	0.4132	0.5136	0.8063
Liaoning	0.6924	0.9590	0.7175	Chongqing	0.5482	0.6448	0.8503
Jilin	0.5808	0.7216	0.7942	Sichuan	0.6869	0.7527	0.9156
Heilongjiang	0.6738	0.8418	0.7953	Guizhou	0.4957	0.5894	0.8449
Shanghai	0.5255	0.5255	1.0000	Yunnan	0.5450	0.5854	0.9347
Jiangsu	0.7844	0.7844	1.0000	Tibet	0.1902	0.2571	0.7463
Zhejiang	0.6756	0.8204	0.8230	Shaanxi	0.5327	0.7826	0.6814
Anhui	0.6218	0.6234	0.9977	Gansu	0.5192	0.6101	0.8524
Fujian	0.5919	0.6755	0.8758	Qinghai	0.2638	0.3972	0.6643
Jiangxi	0.6196	0.6196	1.0000	Ningxia	0.4305	0.5727	0.7532
Shandong	0.9546	0.9803	0.9738	Xinjiang	0.4882	0.7179	0.6838
Henan	0.7939	0.7963	0.9968	Mean	0.5775	0.6840	0.8401

From the perspective of the resource allocation stage, the resource allocation efficiency of the national health system is 0.6840, which still has a lot of room for improvement. Among them, Shandong, Liaoning, Shanxi, Hunan, Heilongjiang and Zhejiang have higher resource allocation efficiency of their health systems, all of which are >0.8, indicating that the health resources in these areas have been effectively allocated, and the medical and health expenditures have achieved a reasonable output level.

From the perspective of the service operation stage, the service operation efficiency of the national health system is high, and the efficiency value reaches 0.84. It can be seen that the service operation of China's health system is relatively good. The empirical results of Yu et al. (77) also showed that the overall efficiency of China's medical and health service system was relatively high from 2010 to 2017, and it was found that after excluding the influence of the environment and random error factors, the overall efficiency of China's medical service system was mostly in the stage of increasing scale. It can be seen that the conclusion of this paper is consistent with the relevant literature. Among them, the efficiency values of Shanghai, Jiangsu, Guangdong, and Jiangxi are all 1, reaching the optimal production frontier. This indicates that the service quality of the health system in these areas is high and the scale effect of health output can be achieved.

#### Evolution trend of health system efficiency in China

**Figure 2:**
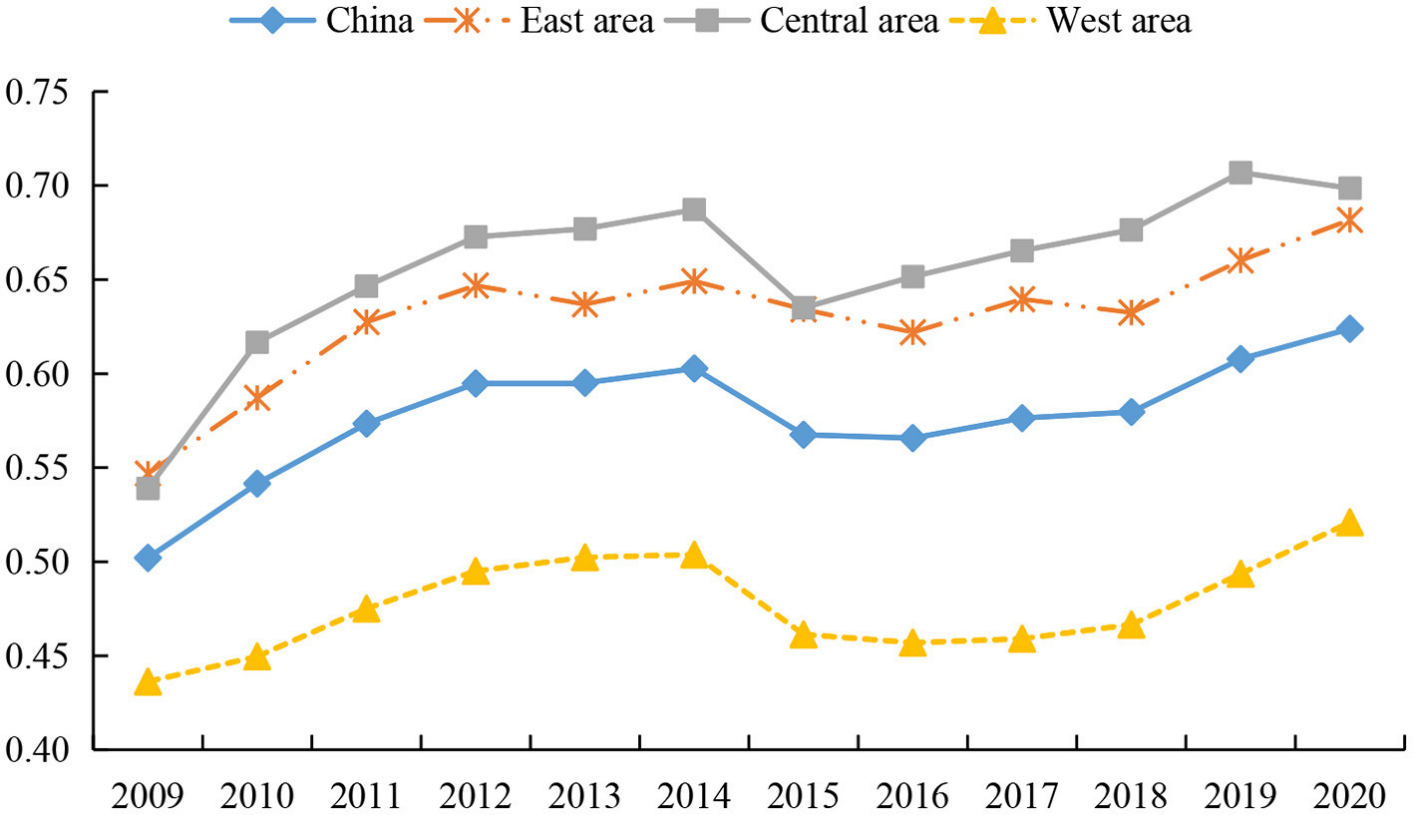
Trends of overall health system efficiency in different regions from 2009 to 2020.

According to the economic development level and geographical location of different regions, we divided the samples into eastern, central and western regions. The eastern region includes Beijing, Tianjin, Hebei, Liaoning, Shanghai, Jiangsu, Zhejiang, Fujian, Shandong, Guangdong and Hainan. These provinces and cities are mainly located along the coast and are the most developed regions in China. The central region includes Shanxi, Jilin, Heilongjiang, Anhui, Jiangxi, Henan, Hubei and Hunan. And the western region includes Inner Mongolia, Guangxi, Chongqing, Sichuan, Guizhou, Yunnan, Tibet, Shaanxi, Gansu, Qinghai, Ningxia and Xinjiang, which are the least developed regions. We draw the trend chart of their overall efficiency of health systems, as shown in Figure 2. As can be seen, the overall efficiency of the national health system exhibits a fluctuating upward trend, rising from 0.5020 in 2009 to 0.6239 in 2020. There are divergent views in the existing literature on trends in health system efficiency. Some scholars believe that the efficiency of China's health system shows a slight downward trend (78, 79). A few studies believe that the efficiency of China's health system has an irregular evolution trend. For example, Zhou (39) emphasized that the overall efficiency of the Chinese government's health spending from 2008 to 2018 was almost an “Ω”-shaped fluctuation trend. However, the opinions of Yu et al. (9) are consistent with the conclusions of this study. We all believe that the overall efficiency of China's health system has shown a fluctuating upward trend after 2009.

As can be seen from Figure 2, the overall efficiency of the health system increased significantly from 2009 to 2014, which mainly relates to the reform of China's medical and health system in 2009. Local governments continue to improve the quantity and quality of the supply of health resources, standardize the order of drug circulation, and constantly improve the operating mechanism of health institutions, thereby promoting the rapid improvement of health level (9). From 2014 to 2015, the overall efficiency of the health system declined briefly, which may be due to the large investment of health resources in the few years before and the rapid improvement of service quality of large-scale health institutions, resulting in an influx of a large number of health technicians and patients into large-scale health institutions. This not only leads to the continuous loss of talents in grassroots health institutions, but also fails to make effective use of grassroots medical resources, thus reducing the operation efficiency of the health system (65). However, the overall efficiency of the health system recovered steadily from 2016 to 2020, as local governments began to pay attention to improving the benefit compensation mechanism, which in turn helps to improve the performance of the health system.

In terms of sub-regions, the overall efficiency of the health system in the eastern, central, and western regions shows an upward trend, and there are great differences in the efficiency of the health system among them. The overall efficiency of the health system in the eastern and central regions is higher than the national average, while that in the western region is lower than the national average. There is no consensus among scholars on the overall efficiency of health systems in different regions. Most studies have confirmed that the health system efficiency in the eastern region is the highest, and its health system operation is better than that in the central and western regions (77, 80). However, a few scholars believe that the health system efficiency in the eastern region is much lower than that in the central and western regions (79). Although existing studies hold different views on the changing trends and overall levels of health system efficiency in different regions of China, they all agree that there are significant regional differences in China's health system efficiency (77, 81, 82), which is the same conclusion as this paper.

**Figure 3:**
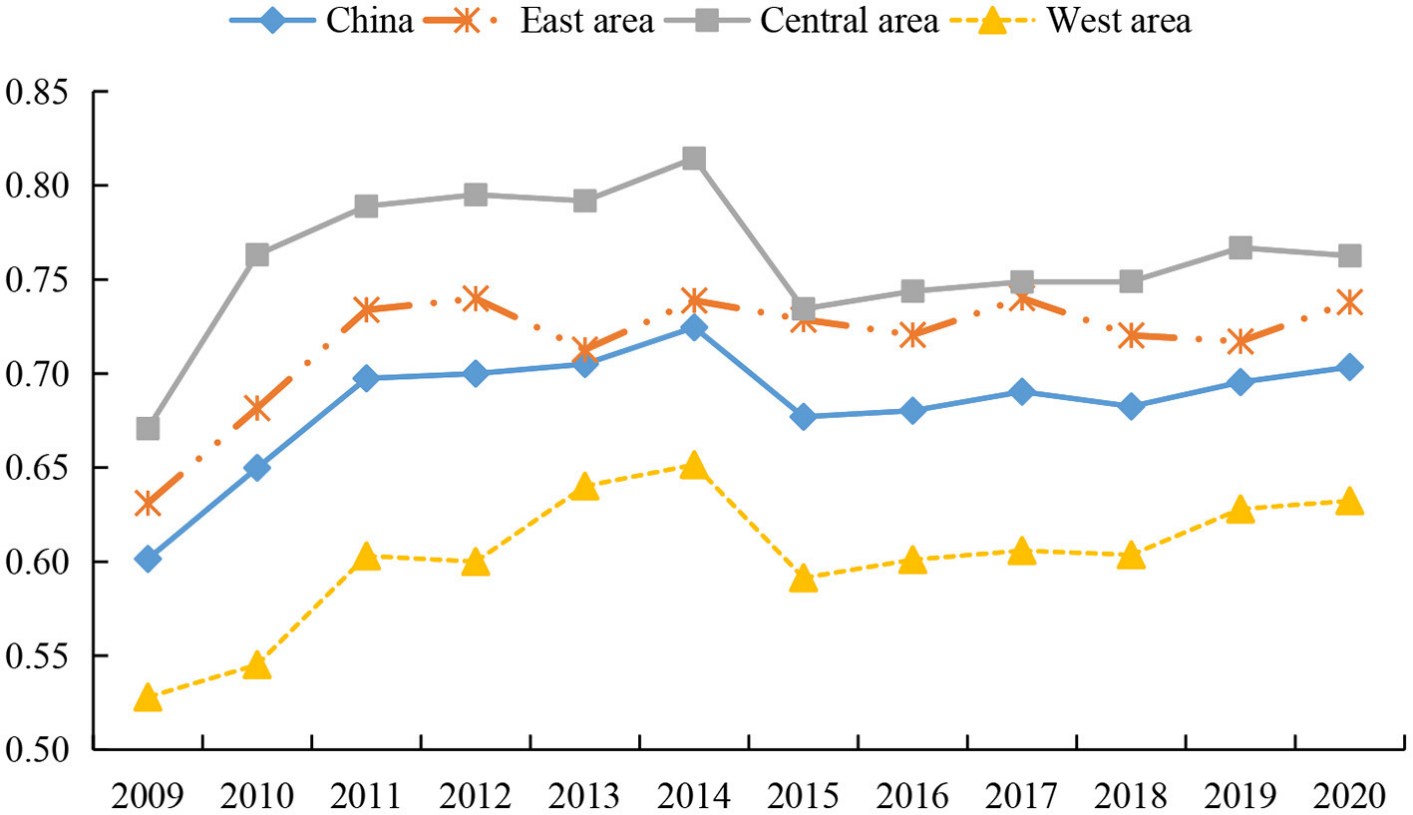
Trends of resource allocation efficiency of health systems in different regions from 2009 to 2020.

**Figure 4:**
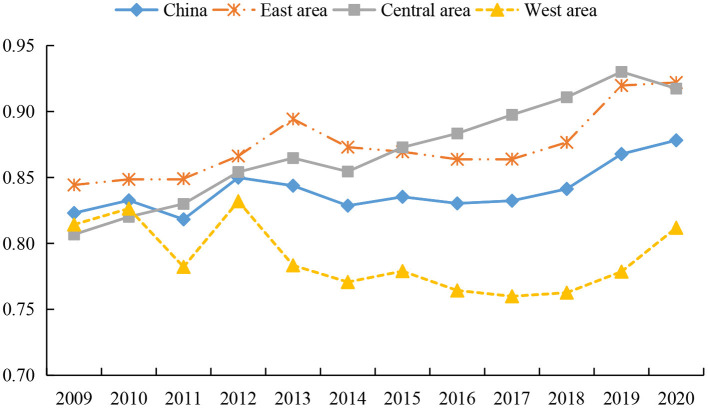
Trends of service operation efficiency of health systems in different regions from 2009 to 2020.

According to the two-stage measurement results, we further analyze the change trend of health system efficiency in the resource allocation stage and service operation stage, as presented in Figures 3, 4. Figure 3 depicts the changing characteristics of resource allocation efficiency of health systems in different regions of China. First, the resource allocation efficiency of health systems in different regions shows a fluctuating and slowly rising trend, which is similar to the conclusions of most studies (76, 79). Among them, the efficiency of health systems in all regions increased significantly from 2009 to 2014, decreased from 2014 to 2015, and then showed a slow upward trend. The reason for this phenomenon is similar to the change of overall efficiency. However, we find that the declining point in the eastern region occurred in 2012. This is due to the fact that there are many large health institutions in the eastern region, and the large investment in health resources in the early stage makes the maintenance cost of medical equipment rise faster, and so the time point of weak growth in resource allocation efficiency appears earlier.

Second, the rankings of the resource allocation efficiency of the health system do not change during the investigation period, that is - the efficiency value of the central region is the highest, followed by the eastern region, and the western region is the lowest. Scholars have different views on the allocation of health resources in different regions. Most studies examine the allocation efficiency of health resources by measuring the health expenditure efficiency, and draw inconsistent conclusions. Some studies emphasize that the improvement efficiency of health resources in the eastern region is the highest (79), while some studies believe that due to the siphon effect, the resource allocation efficiency of the developed eastern region is lower than that of the central and western regions (65). However, we found that most of the studies confirmed that the health resource allocation in the central region is better, and some studies pointed out that the health resource allocation efficiency in the central region is the highest (65, 81, 83), which is similar to the viewpoint of this paper. The explanation of the conclusions of this study can be summarized as follows. The main reason is that the eastern region attracts a large number of floating population due to its high level of economic development and obvious regional advantages, which make the total demand for medical and health care constantly increase. In order to meet the medical needs of a large population, health institutions in the eastern region continue to increase medical equipment and beds. However, due to the lack of supporting management mechanism, a large number of health expenses is only used to maintain the daily operations of health institutions, leading to low efficiency of health technology and a low level of resource allocation (65). The resource allocation efficiency of health systems in the western region is at a low level, which may be restricted by factors such as economic development and geographical location. The input of health cost in the western region is small, but the cost of health resource acquisition and transportation is high, and so high output levels cannot be achieved. Moreover, it is difficult for the backward western regions to attract high-quality health technicians, thereby reducing the allocation efficiency of health resources.

Figure 4 shows the changing characteristics of service operation efficiency of the health systems in different regions of China. First, during the whole investigation period the eastern and central regions showed a fluctuating upward trend, while the western region showed a fluctuating downward trend first and then a rising trend. We see that the service operation efficiency of the health system in various regions increased rapidly from 2011 to 2012. This is explained by the fact that the reform of the medical system in 2009 led to a continuous increase in the input of health resources, while the lag of health output has made the service operation effect in some regions only manifest itself after 2011.

Second, there is little difference between the efficiency values of the eastern region and the central region, while the efficiency values of the western region are at the lowest level, with values of 0.8742, 0.8702 and 0.7888, respectively. For the comparison between the efficiency of the central and western regions, there are differences in the relevant studies. Some scholars believe that during the operation stage of health services, the efficiency of the central region has been lower than that of the eastern and western regions over the years, showing the phenomenon of “central collapse” (65, 68). However, the conclusion about the highest efficiency of health services in the eastern region is consistent with the views of most studies (65, 68, 79). This is because the eastern region not only has advanced medical equipment and high-quality health technicians, but also residents in the region have high health awareness, which makes the service operation efficiency of the health system hit a high level. However, due to fierce market competition in the eastern region, it also causes greater work pressure and work intensity. This situation increases the possibility of residents' illness, brings challenges to the medical service system (39), and reduces the operation efficiency of the health system to a certain extent. Affected by the shortage of financial funds, the western region has insufficient investment in high-quality medical equipment and health technicians, which restrict the improvement of service operation efficiency (82). In addition, a large number of people in the western region flows to economically developed areas, making the existing medical equipment underutilized and a large number of health resources idle and thus reducing the service efficiency of the health system.

#### Temporal and spatial pattern of health system efficiency in China

**Figure 5:**
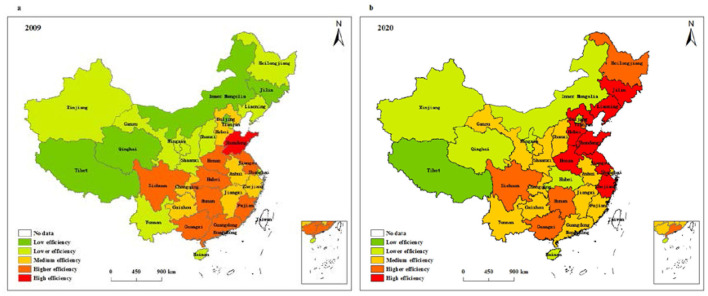
Spatial pattern of overall health system efficiency in **(a)** 2009 and **(b)** 2020.

We further use ArcGIS software to analyze the temporal and spatial pattern of health system efficiency in China. Based on the data of two time points in 2009 and 2020, this study employs the natural fracture method to divide health system efficiency into five types, low efficiency, lower efficiency, medium efficiency, higher efficiency, and high efficiency, so as to explore the spatial evolution characteristics of the health system efficiency in China. Figure 5 displays the spatial evolution characteristics of overall efficiency of the health system in different provinces of China. Accordingly, the regions with low overall efficiency in 2009 include Tibet, Qinghai, Inner Mongolia, Jilin and Beijing, and the regions with high overall efficiency are only distributed in Shandong. In 2020, the regions with low overall efficiency are only distributed in Tibet, and the regions with high overall efficiency are mainly concentrated in eastern regions such as Shandong, Zhejiang, Jiangsu, Hebei, and Liaoning. Compared with 2009, the proportion of regions with medium and above efficiency level has increased significantly, from 48% in 2009 to 74% in 2020. Among them, the areas with high overall efficiency increased significantly, while the areas with low overall efficiency decreased.

**Figure 6:**
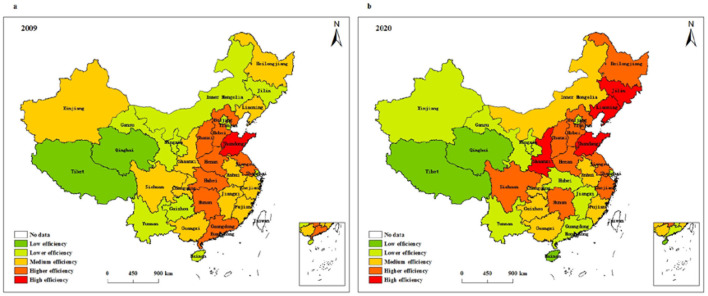
Spatial pattern of resource allocation efficiency of health systems in **(a)** 2009 and **(b)** 2020.

Figure 6 depicts the spatial evolution characteristics of resource allocation efficiency of health systems in different provinces of China. It can be seen from the figure that the regions with low resource allocation efficiency in 2009 include Tibet and Qinghai. The regions with high resource allocation efficiency are only distributed in Shandong, and the regions with higher resource allocation efficiency are concentrated in seven regions, which include Jiangsu, Guangdong, Hebei, Hubei, and Hunan. In 2020, Tibet, Qinghai, and Hainan are regions with low resource allocation efficiency, Shandong, Liaoning, Jilin and Shaanxi are regions with high resource allocation efficiency, and those with higher resource allocation efficiency include nine regions, such as Jiangsu, Zhejiang, Tianjin, Hebei, and Hunan. Compared with 2009, the number of regions with high resource allocation efficiency and higher resource allocation efficiency has increased, while the number of regions with low resource allocation efficiency has hardly changed.

**Figure 7:**
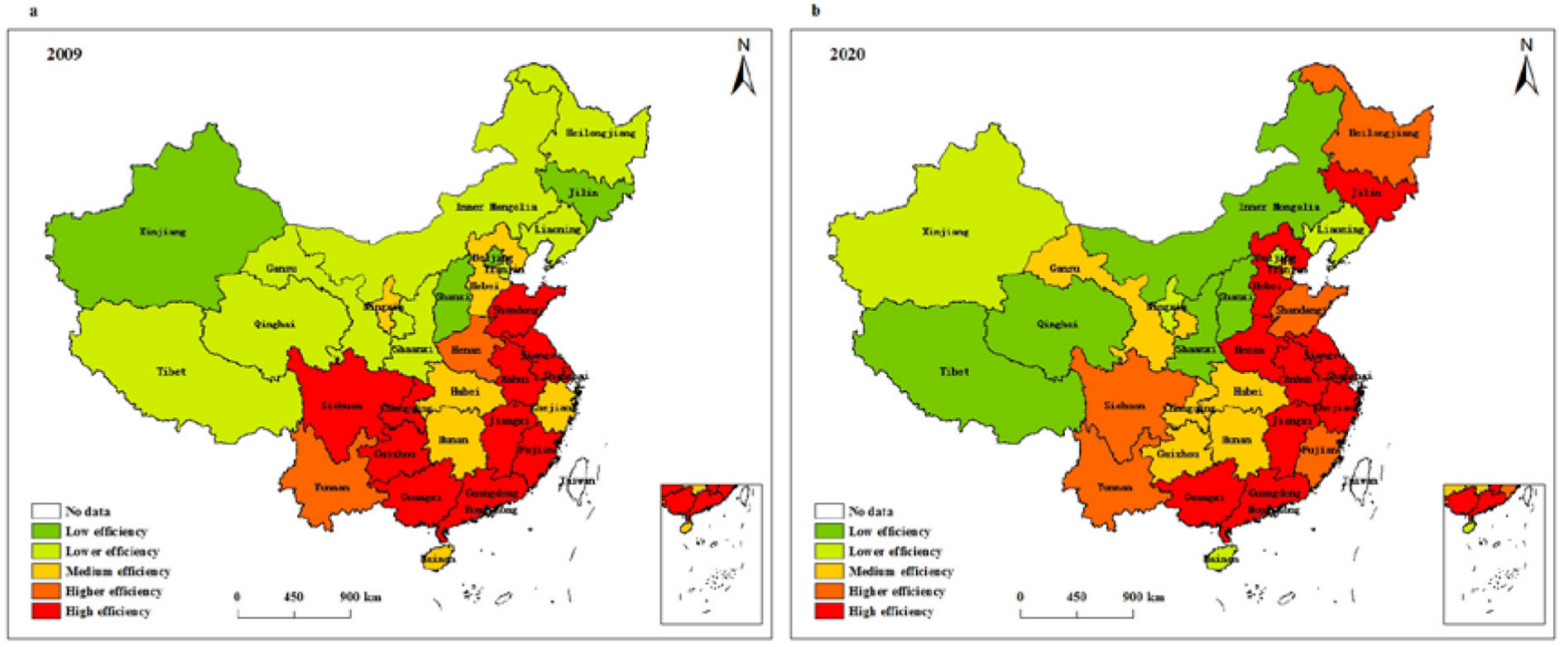
Spatial pattern of service operation efficiency of health systems in **(a)** 2009 and **(b)** 2020.

Figure 7 presents the spatial evolution characteristics of service operation efficiency of health systems in different provinces of China. According to Figure 7, the regions with low service operation efficiency are concentrated in Xinjiang, Shanxi, Jilin, and Beijing, and the regions with high service operation efficiency are mainly distributed in 11 regions, such as Shanghai, Guangdong, Fujian, Anhui, and Jiangsu. In 2020, Tibet, Qinghai, Inner Mongolia, Shanxi, and Shaanxi are regions with low service operation efficiency, and ten regions with high service operation efficiency are mainly distributed in Shanghai, Guangdong, Zhejiang, Hebei, and Henan. Compared with 2009, the number of areas with high service operation efficiency and areas with low service operation efficiency changed little. Moreover, the regions with high service operation efficiency are gradually concentrated from a relatively discrete distribution to the eastern region.

### Analysis of spatial spillover effects of health system efficiency in China

#### Spatial autocorrelation test

The spatial correlation of health system efficiency needs to be examined prior to spatial econometric model analysis. Based on the spatial adjacency weight matrix, this research adopts GeaDa software to calculate the global Moran's *I* index from 2009 to 2020, and the results are in Table 5. As can be seen from the table, the Moran's *I* index of health system efficiency in all years except 2018 passed the 5% significance test, which indicates that the efficiency of provincial health systems in China has significant spatial correlation. From 2009 to 2015, the global Moran's *I* index decreased from 0.3943 to 0.2063, indicating that the spatial correlation of health system efficiency tended to weaken. The global Moran's *I* index fluctuates in the range of 0.1598–0.2227 from 2015 to 2020, and the spatial impact of health system efficiency still persists. In general, the efficiency of China's health system exhibits spatial dependence, that is - the level of health systems in one region affects the level of health systems in adjacent regions through spatial spillover effects.

**Table 5:** Global Moran's *I* index of health system efficiency in China from 2009 to 2020.

**Year**	**Moran's *I***	***Z*-value**	***P*-value**	**Year**	**Moran's *I***	***Z*-value**	***P*-value**
2009	0.3943	3.5778	0.001	2015	0.2063	2.0840	0.026
2010	0.3867	3.5776	0.001	2016	0.2202	2.1932	0.019
2011	0.3126	2.9584	0.002	2017	0.1925	1.9580	0.031
2012	0.2570	2.5132	0.014	2018	0.1598	1.6975	0.057
2013	0.2281	2.2875	0.019	2019	0.1788	1.8455	0.043
2014	0.2224	2.2300	0.018	2020	0.2227	2.1422	0.020

#### Test and identification of spatial econometric models

Due to the significant spatial correlation of health system efficiency, the traditional least squares regression will lead to biased estimation results. Therefore, this study introduces a spatial econometric model to analyze the influencing factors of health system efficiency. Before model estimation, the spatial econometric model needs to be identified and tested. The results appear in Table 6. Generally, the LM test method is used to determine whether SEM or SLM should be selected. Table 6 reports that both LM-spatial error and Robust LM-spatial error pass the significance test at the 1% statistical level, LM-spatial lag is also significant at the 1% statistical level, and Robust LM-spatial lag passes the 10% significance test, indicating that both models of SEM and SLM can be used for empirical test. However, the LM test method does not take into account the applicability of SDM, and so it is necessary to employ the LR test and the Wald test to judge whether SDM will be simplified to SEM or SLM. Table 6 presents that both LR-spatial error and LR-spatial lag reject the null hypothesis at the 1% statistical level, and both Wald-spatial error and Wald-spatial lag also reject the null hypothesis at the 1% statistical level. The results show that SDM cannot be simplified into SEM and SLM. In addition, according to the results of the Hausman test, the index is 48.45 and passes the significance test at the 1% statistical level, which means that the research model should use fixed effects. Therefore, we choose the fixed-effect SDM to empirically investigate the spatial spillover effect of health system efficiency.

**Table 6:** The results of identification tests of spatial econometric models.

**Content**	**Method**	**Statistics**	***P*-value**
Test of SEM and SLM	LM-spatial error	47.67	<0.001
	Robust LM-spatial error	31.87	<0.001
	LM-spatial lag	18.54	<0.001
	Robust LM-spatial lag	2.74	0.098
Simplified test for SDM	LR-spatial error	23.82	<0.001
	Wald-spatial error	24.40	<0.001
	LR-spatial lag	24.16	<0.001
	Wald-spatial lag	25.09	<0.001
Hausman		48.45	<0.001

#### Spatial spillover effects and decomposition results

On the basis of the above analysis, we apply Stata software to conduct a regression test on the factors influencing health system efficiency in 31 provinces in China from 2009 to 2020. The estimated results are in Table 7. As can be seen from the table, the level of economic development, fiscal decentralization, and old-age dependency ratio all pass the significance test at the 1% level. The level of economic development and fiscal decentralization system have a significantly negative impact on health system efficiency, and there exists a significantly positive relationship between old-age dependency ratio and health system efficiency. Since SDM considers the influence of related variables in adjacent areas, the coefficients of the spatial lag terms do not fully explain the actual effects of the variables. Systematic bias will occur if we use the coefficients only to analyze the spillover effect of the variables (40). Therefore, we further decompose the research model into direct effect, indirect effect, and total effect, which can reduce estimation bias to some extent and improve the accuracy of model estimation. The results are in Table 8.

**Table 7:** Regression results of the spatial Durbin model.

**Variable**	**Spatial Dubin model**				
	**Coefficient**	**Standard error**	**z**	**[95% confidence interval]**	
ln*pgdp*	−0.6591***	0.1849	−3.56	−1.0216	−0.2967
ln*fiscal*	−0.3021***	0.0964	−3.14	−0.4910	−0.1132
ln*urban*	−0.1810	0.1740	−1.04	−0.5220	0.1600
ln*edu*	−0.0374	0.0414	−0.90	−0.1185	0.0437
ln*depend*	0.2649***	0.0720	3.68	0.1237	0.4061
W*ln*pgdp*	1.2023***	0.3293	3.65	0.5570	1.8476
W*ln*fiscal*	−0.5274***	0.1843	−2.86	−0.8887	−0.1661
W*ln*urban*	−0.3109	0.3596	−0.86	−1.0158	0.3939
W*ln*edu*	−0.1084	0.0949	−1.14	−0.2944	0.0776
W*ln*depend*	0.3732**	0.1449	2.58	0.0893	0.6572
sigma2_e	0.0097***	0.0007	13.55	0.0083	0.0111
Individual effect	Control				
Time effect	Control				
Observations	372				
Log-likelihood	333.7146				

**Table 8:** Direct, indirect, and total effects of the spatial Durbin model.

**Variable**	**Direct effect**	**Indirect effect**	**Total effect**
ln*pgdp*	−0.7022*** (−3.68)	1.1840*** (4.10)	0.4818 (1.49)
ln*fiscal*	−0.2856*** (−3.17)	−0.4215** (-2.58)	−0.7072*** (−4.05)
ln*urban*	−0.1785 (−1.02)	−0.2546 (−0.78)	−0.4331 (−1.19)
ln*edu*	−0.0289 (−0.73)	−0.0923 (−1.16)	−0.1212 (−1.39)
ln*depend*	0.2505*** (3.60)	0.3039** (2.42)	0.5544*** (4.02)

As far as the direct effects are concerned, the regression coefficient of the level of economic development is significantly negative at the 1% level, meaning that its improvement has a negative impact on local health system efficiency. As for the relationship between the economic development level and the health system efficiency, there are different views in the existing literature. Some studies have pointed out that economic development improves the income level of residents and the supply of health resources, which in turn has a positive effect on the health system efficiency (15, 81, 84). However, some studies believe that the high growth of the regional economy has inhibited the improvement of the health system efficiency (39). The research in this paper confirms the latter viewpoint again. This may be due to the siphoning effect of higher levels of economic development on the labor force (85). A large amount of labor force flows from economically backward regions to developed regions, resulting in excessive population density and an overload of the health system in economically developed regions, which in turn inhibits the service efficiency of these health systems.

The regression coefficient of fiscal decentralization is significantly negative at the 1% level, which confirms that a fiscal decentralization system reduces health system efficiency in the region. Although some studies suggest that fiscal decentralization can improve the health system efficiency by making the government's health spending policy more flexible (78), most studies affirm the negative impact of fiscal decentralization on the health system efficiency (39, 81, 82). Possible explanations for this result are as follows. On the one hand, a fiscal decentralization system enables local governments to have greater power over resource allocation (86). Under the motivation of “promotion tournament,” local governments are more inclined to invest funds in infrastructure construction while ignoring the improvement of people's livelihood in order to pursue high GDP growth (39). The inefficiency of a local health system is mainly caused by the lack of attention and investment of the local government in the health sector (87). On the other hand, the long-term tax-sharing system widens the financial gap between regions, restricts the supply capacity of the health system in the regions with backward financial resources, and then reduces the operation efficiency of the health system (88).

The regression coefficient of the old-age dependency ratio is significantly positive at the 1% level as well. This result is different from the general conclusion. Existing studies generally believe that the aging population aggravates the shortage of health resources, which seriously hinders the improvement of health system efficiency (15, 84). However, the reasons for the conclusion of this study can be summarized as follows. This may be due to the fact that China is still in the early stage of population aging, and the negative effect of the increase in the proportion of old-age dependency on the health system efficiency has not yet arisen (39). In the early stage of population aging, due to the deterioration of the physical functions of the elderly population, the total demand for public medical resources gradually increases, which promotes the improvement of medical resource supply and service capacity in the region to a certain extent. As the aging of the population becomes increasingly prominent, local governments start to pay attention to investment in elderly services, especially in medical and health care (89), thus helping to improve the service efficiency of the health system.

The level of urbanization has a negative effect on health system efficiency, but the result is not significant. By comparing other studies, it is found that some studies emphasize that urbanization means higher residents' income, better infrastructure and medical services, which is conducive to improving residents' medical treatment level and driving the improvement of health efficiency (9, 81). However, with the acceleration of China's urbanization process, the negative effect of urbanization on the health system efficiency has become increasingly prominent (78, 90). We believe that the reason for its negative effect is related to the siphon effect of the labor force, which is similar to the mechanism of economic development level. The illiteracy rate has a negative impact on health system efficiency, but the regression results are also insignificant. The reason for the negative correlation can be explained as the improvement of education level helps to improve the professional quality of health technicians and also enhances the health consciousness of residents, thus contributing to the improvement of the service efficiency of the health system.

The regression results of indirect effects suggest that the coefficient of economic development level is significantly positive, indicating that the improvement of economic development level in an adjacent region will promote the improvement of local health system efficiency. Possible explanations for this result are as follows. On the one hand, the developed economic level of adjacent areas drives the rapid development of the local economy and the improvement of people's livelihood through technological spillovers and trade exchanges, which are conducive to improving the supply capacity of local health resources. On the other hand, adjacent areas with a higher economic level may promote labor force migration to the local area, resulting in the aggregation and increase of human capital and thereby helping to improve the service efficiency of the local health system. Moreover, the cross-regional mobility of labor forces will also promote the full utilization of local medical resources. The regression coefficient of fiscal decentralization is significantly negative at the 5% statistical level, which shows that the degree of fiscal decentralization in an adjacent region inhibits the improvement of local health system efficiency. This is mainly due to the fact that the higher the degree of fiscal decentralization is in an adjacent region, the more likely the adjacent governments is to promote economic development by distorting fiscal supply decisions. The existence of competition between regions makes local governments follow the practice of an adjacent region, that is - an increase in investment in infrastructure construction at the expense of financial resources in health care and other livelihood areas leads to a serious shortage of service supply in the local health system and ultimately reduces the operating efficiency of local health system.

The regression coefficient of the old-age dependency ratio is significantly positive at the 5% statistical level, which suggests that an increase of the old-age dependency ratio in an adjacent region promotes the efficiency of the local health system. The reason may be that large numbers of elderly people in adjacent areas have increasing demand for public medical resources, which will prompt some elderly people in adjacent areas to turn to local health institutions for medical treatment, thereby driving local health resources to be fully utilized. The level of urbanization fails to pass the significance test, indicating that the impact of urbanization on health system efficiency does not have a spatial spillover effect. There is also no spatial spillover effect between illiteracy rate and health system efficiency, which may be due to the investment in educational resources in adjacent areas being independent of each other, the total migration of high-quality talents from adjacent areas to the local area is small, and the service efficiency of the local health system has not been greatly improved.

## Conclusion and policy recommendations

Based on panel data of 31 provinces in China from 2009 to 2020, this research adopts the two-stage network DEA model to provide a comprehensive measurement of health system efficiency. On this basis, the spatial econometric model is used to deeply explore the influencing factors and spatial spillover characteristics of health system efficiency throughout China.

The findings in this paper mainly include the following aspects. First, the overall efficiency of China's health system is low, which is mainly caused by the low efficiency of resource allocation. Specifically, health system services are operating relatively well, while resource allocation still has room for improvement. Second, there are differences in the evolution of trends in health system efficiency. The overall efficiency of China's health system show a fluctuating upward trend, with large differences between different regions. From the perspective of the resource allocation stage, the resource allocation efficiency of health systems in different regions fluctuates and rises slowly. Among them, the central region has the highest efficiency value, followed by the eastern region, and the western region has the lowest efficiency value. From the perspective of service operation efficiency, the eastern and central regions shows a fluctuating upward trend, while the western region shows a fluctuating downward trend first and then rising trend. Among them, the efficiency value of the eastern region is similar to that of the central region, and the efficiency value of the western region is at the lowest level. Finally, the improvement of economic development level and fiscal decentralization significantly reduces health system efficiency, and an increase of the old-age dependency ratio promotes the development of the health system. In terms of spatial spillover effects, the improvement of economic development level and old-age dependency ratio in adjacent regions can help the efficiency of the local health system, while an increase in the degree of fiscal decentralization in adjacent regions hinders the improvement of the efficiency of the local health system. There is no spatial spillover effect of urbanization and illiteracy rate on health system efficiency.

Based on the above discussion, we put forward the following policy recommendation.

First, the government should pay attention to regional disparities in health system efficiency and allocate medical resources rationally. On the one hand, the investment scale of health resources should be adjusted according to the economic strength and resource endowment of different regions. Specifically, the eastern region should implement refined health resource management and improve the medical management system to avoid wasting resources due to redundant inputs. The government should also strengthen policy support to the medical system in the western region and continue to increase financial investment. On the other hand, cooperation among health institutions in different regions can be enhanced to improve the regional imbalance of China's health system. Especially in the post-epidemic era, the government should establish a joint prevention and control mechanism for major risks and improve information sharing and health resource deployment capabilities between regions, so as to achieve precise and effective prevention and control of epidemics. An important way to improve efficiency is to establish the mechanism of supporting health resources in backward areas, such as the sharing of health resources and the construction of medical alliances.

Second, to improve the technical level and management capacity of the input elements of health resources, it is suggested that health institutions carry out business training, special training, and domestic and foreign training programs to improve the professional skills of health technicians. At the same time, health institutions need to introduce advanced medical equipment and management models to promote the rapid improvement of the service efficiency of the health system. In addition, local governments should encourage technological innovation in the medical field and increase investment in medical technique research and development, so as to continuously improve the health status of patients.

Third, local governments need to strengthen and improve the economic and institutional environments that affect the efficiency of health systems. In terms of the economic development environment, local governments should guide high-quality resources in developed areas to gather in backward areas and continuously improve the economic development and health service system of backward areas, so as to reduce the burden on the health system in developed areas. In the aspect of institutional environment construction, the government must focus on constraining investment preferences in production and construction and increase the proportion of public service provision in government assessment, especially in the assessment of health system quality. Moreover, the government should adhere to the matching of powers and expenditure responsibilities, so as to avoid excessive sinking of expenditure responsibilities. In terms of helping the elderly population, the government needs to formulate health service policies related to this growing population and increase the supply of health service facilities for the elderly. In the education field, governments must emphasize the health education of residents and strengthen the popularization of health knowledge, which can then promote the improvement of health system efficiency.

## Data availability statement

The original contributions presented in the study are included in the article/supplementary material, further inquiries can be directed to the corresponding author/s.

## Author contributions

Conceptualization, writing—original draft preparation, and visualization: YY and LZ. Methodology, software, and resources: YY. Validation and data curation: LZ, YY, and WZ. Formal analysis: XZ. Investigation: MY. Writing—review and editing: WZ and YY. Supervision, project administration, and funding acquisition: WZ. All authors have read and agreed to the published version of the manuscript.

## Funding

This research was funded by the National Social Science Fund General Project of China (No. 19BGL092), Innovation Strategy Research Project of Fujian Province (No. 2021R0156), GF Securities Social Welfare Foundation Teaching and Research Fund for National Finance and Mesoeconomics.

## Conflict of interest

The authors declare that the research was conducted in the absence of any commercial or financial relationships that could be construed as a potential conflict of interest.

## Publisher's note

All claims expressed in this article are solely those of the authors and do not necessarily represent those of their affiliated organizations, or those of the publisher, the editors and the reviewers. Any product that may be evaluated in this article, or claim that may be made by its manufacturer, is not guaranteed or endorsed by the publisher.
